# Genome Sequence of a Diverse Goose Circovirus Recovered from Greylag Goose

**DOI:** 10.1128/genomeA.00767-15

**Published:** 2015-07-30

**Authors:** Tomasz Stenzel, Kata Farkas, Arvind Varsani

**Affiliations:** a Department of Avian Diseases, Faculty of Veterinary Medicine, University of Warmia and Mazury, Olsztyn, Poland; b Biomolecular Interaction Centre and School of Biological Sciences, University of Canterbury, Christchurch, New Zealand; c Department of Clinical Laboratory Sciences, Electron Microscope Unit, University of Cape Town, Cape Town, South Africa; d Department of Plant Pathology and Emerging Pathogens Institute, University of Florida, Gainesville, Florida, USA

## Abstract

A diverse goose circovirus (GoCV) genome was recovered from a wild hunted greylag goose *(Anser anser*) in Poland. The genome shares 83% pairwise identity with other GoCV genomes recovered from various geese from China, Germany, and Taiwan.

## GENOME ANNOUNCEMENT

Circoviruses are small circular DNA viruses (~1.6 to 2 kb), which have an ambisense genomic organization and two large open reading frames. The replication-associated protein (Rep) is encoded on the virion strand, whereas the capsid protein is encoded on the complementary strand. Circoviruses are associated with fatal diseases in a variety of birds (including canaries, ducks, finches, geese, parrots, pigeons, ravens, starlings, and swans), in which infection can lead to lymphoid tissue damage and immunosuppression (1).

Goose circovirus (GoCV) was first identified in a commercial flock with a runting syndrome in Germany in 1997 (2) and subsequently has been identified in other European countries and the Far East. In the 18 years following the discovery of GoCV, 36 full genomes (3–5) have been recovered from geese from China (*n* = 11), Germany (*n* = 1), and Taiwan (*n* = 24), and these genomes share >91% genome-wide pairwise identity.

Here, we identified a divergent GoCV in a wild greylag goose (*Anser anser*) that was hunted by a sport hunter (November 2012) in the area around Gopło Lake (Kujawsko-Pomorskie province in Poland). The area of Gopło Lake is inhabited by a large population of greylag geese, and it is also a resting place of various species of wild geese during the fall migration. The bird was sexually mature, healthy, and probably originated from the local population.

Total DNA was extracted directly from a liquid medium used for transporting cloacal swabs with the magnetic method using the Janus automated workstation (PerkinElmer, USA) and the NucleoMag tissue kit (Macherey-Nagel, Germany), in accordance with the manufacturer’s instructions. The broad-spectrum nested-PCR method targeting the *rep* gene of various avian circoviruses was used as described by Halami et al. (6). Following this, we designed a set of abutting primers (GoCV-PL-F, 5′-CCA GGC TCT TCC TCC CAG CKW CTC TT-3′; and GoCV-PL-R, 5′-CTS TCT CGW GCY CGG GGA TCT GAC-3′) and used these with Kapa HiFi HotStart DNA polymerase (Kapa Biosystems, USA) to recover the full genome. The ~1,700-nucleotide (nt) amplicon was cloned into pJET1.2 vector (Life Technologies, USA) and sequenced by primer walking at Macrogen, Inc. SDT version 1.2 (7) was used to determine the pairwise identities of the genomes and the Rep and CP amino acid sequences.

The 1,794-nt genome of the Polish wild greylag goose GoCV (isolate 2GK) shares 83% genome-wide pairwise identity with the 36 genomes of GoCV available in public databases. The Rep and CP of GoCV 2GK share <92% and <77% pairwise amino acid identity, respectively, with those of other GoCVs. We did not find any evidence of recombination in the GoCV 2GK genome using the recombination detection program (RDP) 4 (8, 9). However, it is likely that recombination plays a significant role in the evolution of GoCVs, similar to that noted for other avian circoviruses (10–14). 

### Nucleotide sequence accession number.

The complete genome sequence of the goose circovirus isolate 2GK has been deposited at GenBank under the accession no. KR869727.
